# Square or Sine: Finding a Waveform with High Success Rate of Eliciting SSVEP

**DOI:** 10.1155/2011/364385

**Published:** 2011-09-15

**Authors:** Fei Teng, Yixin Chen, Aik Min Choong, Scott Gustafson, Christopher Reichley, Pamela Lawhead, Dwight Waddell

**Affiliations:** ^1^Department of Computer and Information Science, University of Mississippi, MS 38677, USA; ^2^Department of Psychology, University of Mississippi, MS 38677, USA; ^3^Department of Health, Exercise Science, and Recreation Management, University of Mississippi, MS 38677, USA

## Abstract

Steady state visual evoked potential (SSVEP) is the brain's natural electrical potential response for visual stimuli at specific frequencies. Using a visual stimulus flashing at some given frequency will entrain the SSVEP at the same frequency, thereby allowing determination of the subject's visual focus. The faster an SSVEP is identified, the higher information transmission rate the system achieves. Thus, an effective stimulus, defined as one with high success rate of eliciting SSVEP and high signal-noise ratio, is desired. Also, researchers observed that harmonic frequencies often appear in the SSVEP at a reduced magnitude. Are the harmonics in the SSVEP elicited by the fundamental stimulating frequency or by the artifacts of the stimuli? In this paper, we compare the SSVEP responses of three periodic stimuli: square wave (with different duty cycles), triangle wave, and sine wave to find an effective stimulus. We also demonstrate the connection between the strength of the harmonics in SSVEP and the type of stimulus.

## 1. Introduction

 A brain-computer interface (BCI) translates brain activities into commands that control external devices. BCI research was initially motivated by the need of a new type of communication tools for paralyzed or elderly people [1, 2]. In recent years, many researchers have investigated BCI for computer gaming and entertainment applications [3–6], which makes noninvasive electroencephalography (EEG) a popular choice [7]. Three types of neuronal signals are most commonly used in EEG-based BCI systems: event-related potentials (ERP) [6, 8, 9], motor-imagery-related brain activity [10, 11], and steady state visual evoked potentials (SSVEP) [12–20].

Among these choices, SSVEP is viewed, by many researchers, as a promising electrophysiological source for BCI systems [21]. When looking at a light stimulus flickering at a given frequency, a user's SSVEP is entrained at the same frequency. Hence, by examining the EEG signal, a simple algorithm can identify the corresponding stimulus at which the subject is looking [22, 23]. It has been reported that SSVEPs can be elicited in the range of 4–100 Hz [24–26]. Although the strongest responses were observed in the range of 5–20 Hz, high-frequency stimuli (greater than 30 Hz) present minimal safety hazards due to photo-induced epileptic seizures [27].

Because EEG is always mixed with background noises, the efficacy of an SSVEP-based BCI system relies heavily on the signal-noise ratio. Intuitively, SSVEP will be detected much faster and with greater easy if the signal-noise ratio is high. The faster an SSVEP is identified, the faster a BCI system can correctly respond, hence a higher information throughput [28]. As it is currently unknown whether the choice of a square wave, triangle wave, or sine wave light signal affects the strength of the elicited SSVEP, these three waveforms (square wave with different duty cycles) were compared in Section 3 for their success rate in eliciting an SSVEP response. In addition, researchers have observed that a stimulus at frequency *f* can elicit SSVEP not only at *f*, but also at harmonics 2*f*, 3*f*, or sometimes even higher orders [29, 30]. This seems to suggest that harmonics may be used to detect the stimulating frequency. In order to take advantage of the harmonics in the design of a BCI system, the following question needs to be addressed: are SSVEP harmonics elicited by the fundamental frequency, that is, *f*, or by the artifacts of the stimulus?

From a signal perspective, the commonly used flickering stimulus is a periodic square wave with 50% duty cycle. Its spectrum contains nonzero Fourier components at ±(2*k* − 1)*f*, *k* = 1,2,…. Therefore, under a square wave stimulus, the cause of a 3*f* harmonic in SSVEP is unclear. In this paper, we explore the SSVEP responses of three periodic stimuli: square waves with different duty cycles, triangle waves, and sine waves. This group of waveforms provides us with the flexibility to adjust the strength of harmonics in a stimulus, hence allowing us to investigate the effects of artifact on SSVEPs.

The remainder of the paper is organized as follows. In Section 2, we describe the stimuli used in the experiments and the experimental setup. The results are given in Section 3. We conclude in Section 4.

## 2. Methodology

Three types of periodic stimuli were used in the experiments: square waves (with duty cycle *τ* ∈ (0,1)), triangle waves, and sine waves. If we define the relative strength of the *k*th harmonic frequency with respect to the fundamental frequency as *r*(*k*) = |*G*_*k*_/*G*_1_|, where *G*_1_ and *G*_*k*_ are the Fourier coefficients for the fundamental frequency and the *k*th harmonic frequency, respectively, it is straightforward to show that *r*_sine_(*k*) = 1 for *k* = ±1 and 0 otherwise; *r*_triangle_(*k*) = [(*π*/2)sine(*kπ*/2)]^2^; *r*_square_(*k*) = |sine(*kτ*)/sine(*τ*)|. Clearly, in theory, there are no harmonic frequencies in a sine wave. In a triangle wave, the harmonic frequencies only exist for odd *k*. Its magnitude is proportional to 1/*k* ^2^. For a square wave with duty cycle *τ* = 0.5, there are also no harmonics for even *k*. The magnitude of odd harmonics is, however, proportional to 1/*k*, that is, stronger than that of a triangle wave. Note that the magnitude of harmonics of a square wave depends on its duty cycle, for example, *r*_sine_(2) > 0 for *τ* ≠ 0.5.

**Figure 1:**
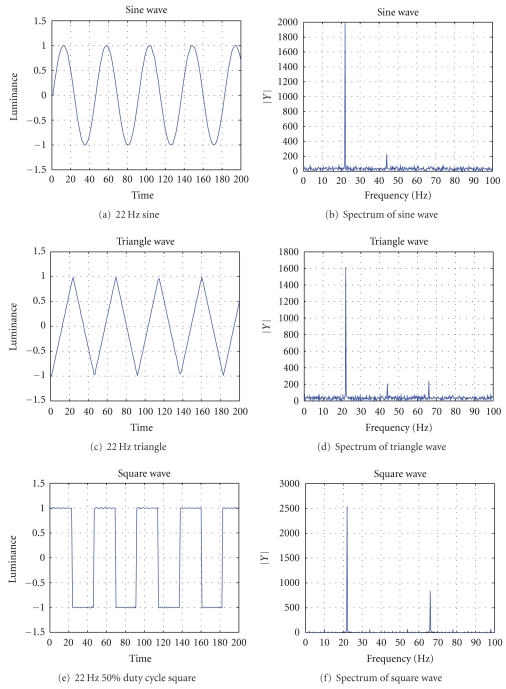
(a), (c), and (e) are the luminance figures of an LED measured by a Lutron LX-102 light meter. Their corresponding frequency representations are given in (b), (d), and (f), respectively. The spectrum of the square wave strictly adheres to theory, that is, a peak demonstrated at fundamental frequency *f* as well as a peak at the 3*f* harmonic. The sine wave and the triangle wave do not. They have weak harmonics that should not exist at 2*f*. However, these harmonics should not affect the result, since their strength are one tenth that of the fundamental frequency.

The above wave forms were rendered using an LED. In order to generate sine and triangle luminance signal, the LED needs to work in a linear (or close to linear) operating region. For the LED used in our experiments, a 3.25 V DC bias was applied. The resulting linear operating region is [3 V, 3.5 V]. The luminance of the LED was converted to an electrical signal using a Lutron LX-102 light meter. The output of the light meter was visualized using a Agilent 54621D oscilloscope and recorded using an integrated sound card.  Figure 1 shows the luminance signal and its spectrum of the three waves. Note that the light signals were not perfectly sine, triangle, or square waves due to the nonlinearity of the LED. The artifacts in the sine and triangle waves were more significant than in the square wave. For example, 2*f*, which should not exist theoretically in sine or triangle waves, appeared in the measured luminance signal. Nevertheless, the amplitude of 2*f* in the measured sine or triangle luminance is roughly one order of magnitude smaller.

Five subjects participated in this experiment. EEG was recorded with one channel over the occipital cortex at a sampling rate of 1 kHz, filtered by a 0.15 Hz high-pass filter and a 150 Hz low-pass filter. The distance between the LED and a subject was 50 cm. We examined stimuli of 11 Hz, 13 Hz, 15 Hz, 18 Hz, and 22 Hz and recorded the SSVEPs of square, triangle, and sine waves. Square waves were generated with 10%, 25%, and 50% duty cycles. In each recording session, the subject was told to look at the stimulus for 8 seconds and close their eyes for a rest period of a random duration from 10 to 20 seconds. The recorded data were discarded when muscle movements artifacts were significant. 

## 3. Results

The primary research goals of these experiments are to find out what kind of waveforms is preferred for future SSVEP based systems.  Table 1 reports the SSVEP results from all subjects. *f* is the fundamental frequency of the stimulus. * “Valid trials”* is the number of trials where the magnitude of FFT coefficients of SSVEP at *f*, 2*f*, or 3*f* are 50% greater than the baseline.* “Total trials"* is the number experiments in which a stimulus is presented to a user regardless of whether the SSVEP peaks were detected. “1*f occurs, *2*f occurs*, and 3*f occurs*” are the number of observed SSVEP peaks at 1*f*, 2*f* and 3*f*, respectively.

**Figure 2:**
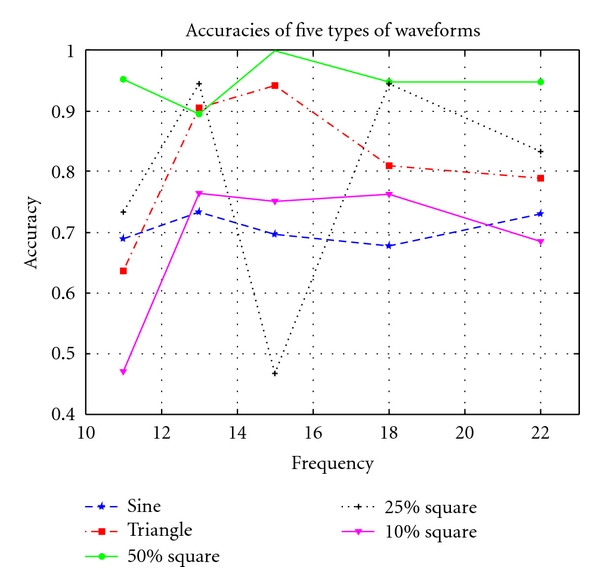
11, 13, 15, 18, and 22 Hz were used as the stimulus frequencies. The accuracies of the SSVEP experiments are computed with equation Accuracy = 1*f*  occurs/Total  trials.

Theoretically, SSVEP peaks appear at the stimulus frequency 1*f* and its harmonics 2*f*, 3*f*, and so forth. An SSVEP system has to use an recognizable 1*f* component to identify which frequency the subject is looking at, while it sometimes uses its harmonics to improve the accuracy. Thus, a valid trial without a 1*f* peak may not be acceptable in a real SSVEP system. So, we define a trial in which 1*f* occurs as an accurate trial, and the accuracy of a certain type of waveform of a certain frequency is Accuracy_wave,frequency_ = 1*f*  occurs/Total  trials.  Figure 2 shows the accuracies of SSVEP trials driven by the three waves above.

We have the following observations.

- *A square waves with *50%* duty cycle have a significantly higher accuracy than other stimuli in our experiment. *As shown in Figure 2, the average accuracies (∑_all  frequencies_number of accurate trials/∑_all  frequencies_total  number of trials) of sine, triangle, and square waves with duty cycle 50%, 25%, and 10% were 70.4%, 81.0%, 94.7%, 79.8%, and 69.1%, respectively. Using statistic analysis techniques, we check if the performance of 50% square wave is better than that of triangle wave, which is intuitively the second best waveform as seen in Figure 2, with a significant level less than 0.05. (90/95) 50% square waves and (81/100) triangle waves evoked 1*f* SSVEP, thus $Z=\left(p_{1}-p_{2})-(\pi_{1}-\pi_{2}\right)/\sqrt{p_{1}(1-p_{1})/n_{1}+p_{2}(1-p_{2})/n_{2}}=1.728$. Since $Z_{\alpha }=(\bar{x}-\mu_{0})/(\sigma /\sqrt{n})=1.645<Z$, we conclude that square waves with 50% duty cycle have a significantly higher accuracy than other stimuli in our experiment.

(ii) *A square wave has a higher success rate than sine or triangle waves in eliciting SSVEPs.* In our experiments, the success rates (number of valid trials divided by the total number of trials) for sine, triangle, and square waves were 75.0%, 83.0%, and 90.8%, respectively.
(iii) *All three wave forms elicited *2*f component in SSVEPs. *In our experiments, the success rates for 2*f* component in SSVEP were 42.9% for sine waves, 48.2% for triangle waves, and 56.2% for square waves (averaged over all three duty cycles). Among the three duty cycles, 10%, 25%, and 50%, of the square wave, the 2*f* success rates were 43.0%, 70.7%, and 59.0%, respectively.

(iv) *A square wave has a significantly higher success rate than sine or triangle wave in eliciting *3*f component in SSVEPs. *In our experiments, the success rates for 3*f* component in SSVEP were 18.4% for sine waves, 14.0% for triangle waves, and 48.0% for square waves (averaged over all three duty cycles). Among the three duty cycles, 10%, 25%, and 50%, of the square wave, the 3*f* success rates were 44.6%, 50.7%, and 55.0%, respectively.

Although sine, triangle, and square waves with 50% duty cycle do not contain 2*f* component, they all elicited 2*f* in SSVEP with similar success rates. Square wave with 25% duty cycle contains a strong 2*f* component. Its 2*f* success rate is significantly higher (70.7%). This suggests that (1) the 2*f* component is primarily elicited by the fundamental frequency and (2) the artifacts in the stimuli increase the success rate of 2*f* in SSVEP. A similar observation is obtained for 3*f*. This seems to suggest that * although the fundamental frequency can elicit harmonics *(2*f and *3*f in our experiments*)* in SSVEP, the success rate of getting harmonics in SSVEPs is positively correlated with the strength of the artifacts in a stimulus.*

## 4. Conclusion

Our results showed that the harmonics associated with SSVEP are elicited both by the fundamental frequency and the artifacts of the stimuli, with the 2*f* component mainly produced by the fundamental frequency and the 3*f* mainly by the artifacts of square waves. At the same time, SSVEP elicited with square waves do not always contain all the artifactual frequency components, for example, 3*f*, and SSVEP with sine waves may have 3*f* harmonics, which is not a part of the stimuli artifacts.

We also observed that square waves with 50% duty cycle have a significantly higher accuracy than other stimuli in our experiment. As a result, the use of square waves with 50% duty cycle is preferred if high 1*f* SSVEP eliciting rate is the goal, while sine waves for SSVEP simulation should be chosen if few harmonic artifacts are wanted.

## Figures and Tables

**Table 1:** Statistic of harmonics in SSVEP.

	1*f* occurs	2*f* occurs	3*f* occurs	Valid trials	Total trials
11 Hz sine	20	10	7	22	29
13 Hz sine	22	9	2	22	30
15 Hz sine	23	8	5	25	33
18 Hz sine	23	9	6	25	34
22 Hz sine	19	12	1	20	26
11 Hz triangle	14	10	4	16	22
13 Hz triangle	19	10	0	19	21
15 Hz triangle	16	5	5	16	17
18 Hz triangle	17	6	2	17	21
22 Hz triangle	15	9	3	15	19
11 Hz 50% square	20	11	15	20	21
13 Hz 50% square	17	5	5	17	19
15 Hz 50% square	17	9	8	16	17
18 Hz 50% square	18	9	8	19	19
22 Hz 50% square	18	9	8	18	19
11 Hz 25% square	11	9	5	11	15
13 Hz 25% square	17	8	6	18	18
15 Hz 25% square	7	7	7	10	15
18 Hz 25% square	17	14	10	18	18
22 Hz 25% square	15	15	10	18	18
11 Hz 10% square	8	9	4	12	17
13 Hz 10% square	13	13	6	17	17
15 Hz 10% square	15	12	11	19	20
18 Hz 10% square	16	9	10	20	21
22 Hz 10% square	13	6	6	15	19
